# Barriers and facilitators to using an app-based tool for suicide safety planning in a psychiatric emergency department: A qualitative descriptive study using the Theoretical Domains Framework and COM-B model

**DOI:** 10.1371/journal.pone.0355517

**Published:** 2026-10-13

**Authors:** Hwayeon Danielle Shin, Keri Durocher, Iman Kassam, Sridevi Kundurthi, John Torous, Gillian Strudwick, Juveria Zaheer

**Affiliations:** 1 Krembil Centre for Neuroinformatics, Centre for Addiction and Mental Health, Toronto, Ontario, Canada; 2 Institute of Health Policy, Management, and Evaluation, University of Toronto, Toronto, Ontario, Canada; 3 Arthur Labatt Family School of Nursing, Western University, London, Ontario, Canada; 4 Department of Psychiatry, Beth Israel Deaconess Medical Center, Harvard Medical School, Boston, Massachusetts, United States of America; 5 Health Outcomes and Performance Evaluation (HOPE) Research Unit, Institute for Mental Health Policy Research, Centre for Addiction and Mental Health, Toronto, Ontario, Canada; 6 Gerald Sheff and Shanitha Kachan Emergency Department, Centre for Addiction and Mental Health, Toronto, Ontario, Canada; 7 Department of Psychiatry, University of Toronto, Toronto, Ontario, Canada; Jawaharlal Institute of Postgraduate Medical Education and Research, INDIA

## Abstract

**Background:**

Emergency departments (EDs) play a crucial role in suicide prevention, with evidenced-based safety planning interventions (SPI) playing an important role. Traditional safety plans, which are often paper based, come with challenges (i.e., not easily accessible, or easy to misplace). Digital safety planning tools offer an alternative mode of intervention delivery, but clinical implementation remains limited. Using behavioural change frameworks, we aim to provide insights into the factors influencing app adoption. These insights will be used as a knowledge base to design behavior change strategies to promote the implementation of a SPI app in a psychiatric ED.

**Methods:**

We used a qualitative descriptive design to conduct semi-structured interviews with clinicians in a psychiatric ED in Toronto, Canada. Participants encompassed all ED staff, irrespective of their SPI app usage. The semi-structured interview guide was developed using the Theoretical Domains Framework (TDF) and the capability (C), opportunity (O), motivation (M) and behavior (B) (COM-B) model. We then used directed content analysis, identifying findings within the TDF and COM-B domains as barriers, facilitators, or both.

**Findings:**

A total of 29 ED clinicians were interviewed, including nurses, psychiatrists, social workers, program assistants, and a pharmacist. The majority had not used the app. We observed strong motivation among clinicians across all disciplines to use the app, and they considered SPI as a high-priority ED care strategy closely linked to their professional identity and responsibility. Anticipated barriers regarding the app as a new mode of SPI delivery in routine practice included: documentation, communication, care efficiency, and patient access to smartphones. Some barriers were attributable to the lack of interoperability between the app and the electronic health record and documentation infrastructure for the app.

**Conclusion:**

We identified key behavioral factors influencing the implementation of the SPI app in the psychiatric ED. As such, we found that tailored strategies that can mitigate barriers by targeting capability, motivation, and opportunity are essential, while facilitators that can sustain implementation should be leveraged. Additionally, ongoing monitoring and adaptation of strategies are necessary, as new barriers may arise or facilitators may weaken over time, impacting the sustained use of the app in the ED. We will use these findings to inform the next phase of this work, which involves co-designing targeted and tailored implementation strategies.

## Background

Suicide is a major public health concern that affects many people, including those living with suicidal thoughts and those grieving suicide deaths [1]. Following the definition provided by Silverman et al., the term suicide-related thoughts and behaviors (SRTB) is used herein as an umbrella term to describe a spectrum of suicidal thoughts and self-injurious behaviors, with or without intent, and with or without fatal outcomes [2]. The intent and motivation for individuals engaging in self-injurious behaviors vary and can evolve, and can fluctuate from moment to moment [3–5]. As such, individuals need to engage in self-management strategies, informal and formal help, and therapies to cope with SRTB.

The emergency department (ED) presents a critical window of opportunity to identify those at risk and to provide suicide prevention care [6]. Suicidal ideation is one common presentation in the ED, and the ED is also often the first point of entry for care for individuals who attempt suicide [7–10]. Moreover, the ED may be the most important setting given recent analysis revealing that the highest prevalence (nearly 40%) of deaths from suicide occurs in individuals with physical health problems [11], and it may be where those patients interact with the health system. It is recommended practice in the ED that patients who are identified as being at risk undergo a thorough assessment and are directed to appropriate referrals and follow-up arrangements. These individuals should also go home with a personalized safety plan, a central, effective intervention for suicide prevention [12,13]. Safety planning intervention (SPI) is an evidence-based intervention that has shown to reduce the risk of suicidal behaviour by up to 43% [12]. SPI has a patient-centered focus and is collaborative in nature [6]. This should not be confused with the outdated practice of ‘contracting for safety’ [6], which is still observed in some clinical settings despite its potential harm [14]. A safety plan is a personalized tool for patients that is developed collaboratively with providers and helps them identify coping strategies, emergency contacts, as well as strategies to keep their environment safe [15]. At our local psychiatric ED in Toronto, Canada, a paper-based SPI is a standard discharge practice for patients who are at risk of suicide but are stable enough to go home after thorough psychiatric assessments and/or appropriate follow-up care arrangements. This means that a safety plan is created after a suicidal crisis has occurred, rather than during intense suicidal thoughts, and is developed when the patient is ready for discharge at clinical discretion.

SPI needs to be improved. Patients often lose paper copies of their safety plans, and some reported feeling uncomfortable reviewing the paper copy of their safety plan in public [16,17]. Similar barriers, particularly the inconvenience of using the paper version of the safety plan, such as how easily it can be lost and its lack of easy modification, have also been reported from clinicians [18,19]. As such, the paper modality of the safety plan implies that it may not be available “at hand” during moments of increased intensity of SRTB. Relatedly, the widespread use of mobile phones introduces a new modality for delivering interventions, which is both acceptable and convenient for many individuals [20–22]. Evidence for the digital modality of SPI has begun to accumulate, supporting its benefits [23]. For example, Rainbow et al showed a significant reduction in suicidal ideation and an increase in suicide-related coping observed over three months among the users of the digital SPI app [24]. Similarly in another study by Barrigón et al, recipients of the digital SPI in the ED showed positive outcomes, including fewer subsequent ED visits related to suicidal behaviors compared to individuals who did not receive the digital SPI [25].

There are numerous digital tools available to augment care and/or support individuals in managing their SRTB like apps, telehealth, and wearable monitoring devices [26–28]. However, a plethora of new digital tools does not always lead to adoption. Shin et al. identified 66 digital suicide prevention interventions implemented in clinical settings, including EDs, and found several barriers to adoption [26]. There were both individual and organizational barriers in clinical settings such as clinicians’ technical competence, motivation and preferences for technology, heavy workload, and limited capacity to troubleshoot technical issues promptly [26]. As such, understanding the barriers and facilitators influencing the adoption of these apps is crucial for successful implementation. Assessing implementation barriers and facilitators is important because successful implementation predetermines the subsequent success of evidence-based interventions in generating their intended benefits [29,30]. In other words, understanding the challenges clinicians face in adopting these technologies is imperative, as these challenges are likely to impact the effectiveness of evidence-based interventions (e.g., SPI), especially as they relate to patient experiences and clinical outcomes during and after their transition from the ED to home.

Previous research has accumulated evidence on various factors impacting the adoption of digital health interventions, including patient preferences, healthcare provider attitudes, organizational support, and technological constraints [31–33]. However, there remains a need for more nuanced insights into the behavioral influences that shape the adoption of digital tools in clinical settings. For example, it is currently unknown how attitudes, knowledge, and the system in which clinicians practice interact to influence behavior [26]. Additionally, there is currently a lack of research on barriers and facilitators to implementing digital tools for specific psychiatric concerns, such as SRTB, particularly in acute care settings like the ED [31,33].

Drawing upon the Behaviour Change Wheel (BCW)’s Capability, Opportunity, Motivation-Behavior (COM-B) model [34,35] and the Theoretical Domains Framework (TDF) [36,37], we sought to explore the behavioral influences underlying the implementation of a SPI app, called Hope By CAMH (i.e., Centre for Addiction and Mental Health) [38]. The COM-B and TDF frameworks are synthesized from 19 and 33 behavior change frameworks, respectively, to explain the complex interplay between individual capabilities, intrinsic and extrinsic motivations, and social and environmental opportunities that influence behavior change [34,36]. The TDF can be seen as an extension of the COM-B as it provides more granular details by expanding six COM-B components (i.e., psychological and physical capability, physical and social opportunity, and autonomic and reflective motivation) into 14 domains of behavior influences. Additionally, the BCW provides guidance on how to develop behaviour change strategies based on the identified behavioural influences [34,35]. Although the COM-B and TDF are popular in implementation science research [39,40], they have yet to be used in digital tools for suicide prevention to inform implementation strategies in the EDs. By using these frameworks, we aimed to demonstrate their utility and relevance. Additionally, applying the COM-B and TDF can help provide comprehensive insights into the factors influencing app adoption that will be used as a knowledge base for the design of theory-informed behavior change strategies for promoting the implementation of SPI apps in clinical practice.

## Methods

### Design

A larger, two-phased study was conducted to develop theory-based strategies for implementing the app into routine clinical flow at a local psychiatric ED in Toronto, Canada. This app, Hope by CAMH, was developed through collaborative efforts between patients, families, clinicians, and researchers at the organization. The “Safety Plan” (i.e., SPI) is the core component of the app, which is intended to be completed collaboratively with clinicians. It includes five items for completion, same as the paper version, as they are based on Stanley and Brown [15]: (1) warning signs, (2) reasons for living, (3) coping strategies, (4) support network, and (5) environmental safety. Compared with the paper template, which is directly aligned with the Stanley and Brown model and requires patients to fill in each item, the app provides toggle options that allow users to select the most applicable answers. Under coping strategies, instead of writing down on paper what works and identifying specific situations where certain strategies may apply, the app provides examples of coping strategies for particular situations. For example, in the app, under “I’m feeling too overwhelmed” (situation), there are over 10 different coping strategies to select, with one example being “Try medication, deep breathing, and relaxation tapes.” The completed safety plan can be shared with trusted members within the patients’ social network, such as family members, but this can only happen if the patients choose to share it using the app’s built-in email function. In addition to SPI, the app provides access to wellness activities (e.g., boxed breathing video, journaling), which can serve as coping strategies. The app also provides a direct 911 emergency line, 988 crisis line, and general information about suicide and its risk factors. S1 File includes both paper and app versions of the SPI.

The app was first launched in 2020 and has been available to the public at no cost since then. As described in the study protocol [41], there were several promotional campaigns with pilot implementation and evaluation activities when the app was first launched. These efforts remained in small pockets within the organization with limited sustainment. Moreover, no systematic implementation efforts have been undertaken to integrate the app into routine clinical workflows across other departments including ED. This was the main rationale for conducting the current study. Complete study methods have been published elsewhere, and more information about the app, the background of its development, as well as previous pilot evaluation findings, can be found in the protocol [41]. The current paper presents the results from the first phase of the study. This phase used a qualitative descriptive study design to assess clinicians’ barriers and facilitators to using the app to complete the SPI in the psychiatric ED before discharging patients to home. Since the goal was to identify clinicians’ behavioural influences, clinicians’ perspectives and experiences were the major focus in this phase to identify barriers and facilitators. We used the consolidated criteria for reporting qualitative research (COREQ) checklist to prepare this manuscript [42].

### Setting

The study took place in a psychiatric ED in Toronto, Canada, which offers 24-hour psychiatric acute care services to those aged 16 years and older. This setting is unique as it is Ontario’s only stand-alone psychiatric ED, providing care to approximately 1,300–1,500 adult patients every month. About 60% of patients in the ED’s Extended Observation Unit are admitted for longer stays, which means patients stay in the ED overnight (or more than 24 hours). The remaining 40% of patients are discharged to home or admitted directly to in-patient units on the same day they present to the ED. Furthermore, there are typically between 12 and 40 patients in the ED waiting to be reassessed in the morning on any given day. This ED has a total of 12 inpatient short-stay beds, and is staffed by multidisciplinary care teams, with nurses (i.e., Registered Nurses and Registered Practical Nurses) comprising the largest proportion of the team. The remaining team members include psychiatrists, program assistants, social workers, and pharmacists. Program assistants are unique in this organization; as part of the interdisciplinary team in the ED, they help the unit run smoothly under clinical staff guidance by supporting patient care, promoting safety, engaging in de-escalation, and maintaining a therapeutic environment. Program assistants in this ED possess a Personal Care Worker Certificate or they are pursuing a further training in a health care discipline. They work closely with clinicians in this ED and are responsible for communicating behavioural observations, assessments, and clients’ responses to interventions to maintain the safety and security of the ED. The ED operates with a shift-based model with 24–7 psychiatrist coverage. During the day shift, this typically involves a team of 6–10 nurses, 3 program assistants, 3–4 staff psychiatrists, with up to 5 residents and medical students, 1–3 social workers, and 1–2 pharmacists.

### Participants and sampling

All ED staff were invited to participate in the interviews, regardless of their usage of the app to support SPI. We explored actual barriers and facilitators for users of the app and explored perceived barriers and facilitators for non-users of the app. All members of the clinical team involved in patient care in the ED were eligible to participate, irrespective of their disciplines. We used both convenience and purposive sampling methodologies. Initially, we sought voluntary participation from all ED staff, then adjusted our sampling approach based on the characteristics of the sample during data collection. Due to lower representation from psychiatrists, we issued a targeted call to them and utilized snowball sampling. We aimed to recruit interview participants to reflect the daily staffing of the ED. Capturing perspectives of all clinicians who make up regular clinical setting was important to understand the full ED care pathway, from triage to discharge, as SPI involves multiple actors at different stages of care. Even if some ED team members, such as program assistants or pharmacists, are not directly involved in SPI, it is still important that all staff are aware that SPI is being offered and delivered, since the ED operates as a highly collaborative team environment. Additionally, we assessed our recruitment process based on data saturation, as determined by the research team involved in data analysis. We defined data saturation [43,44] as the point in which no additional insights emerged from the interviews and all relevant conceptual constructs within the COM-B and TDF domains had been explored, identified and completed.

### Recruitment

After receiving ethics approval from the organization (REB# 2023/078), we circulated emails to all ED staff about the interview opportunity in September 2023. Emails were sent out using an internal email listserv, which included 52 psychiatrists providing regular shift coverage in the ED, along with 111 additional staff members, such as nurses, program assistants, pharmacists, and team leads, working in part-time, full-time, and casual capacities. In addition to email communications, the first author made five visits to the ED between September and November 2023, dedicating either half or full days to make announcements during morning huddles, arrange interviews, and conduct in-person interviews on site. Additionally, this ED serves as a training site for over 150 medical learners, including residents and medical students, who were also eligible to participate. On a daily basis, up to five residents and medical students were present, and they were also invited to participate in interviews. Additionally, we hung posters in high-traffic areas such as washrooms, ED workstations, and staff rooms, as determined by the ED Advanced Practice Nurse. To compensate for participants’ time and the insights they shared, we provided them with a $50 e-gift card. More details on recruitment strategies and lessons learned from recruiting ED clinicians have been published elsewhere [45].

### Procedures

We developed the interview guide based on the COM-B, and the TDF, as well as the previous scoping review findings (S2 File) [26]. Interview questions were designed to identify influences of our target behaviour, which was using the app to complete SPI in the ED prior to discharging patients to home. Additional questions specific to equity considerations were added. Paper copies of the SPI will remain available in the ED for those without access to a smart device; however, even among patients with device access, differing digital literacy skills can create gaps in realizing the benefits of the app. Therefore, we explored clinicians’ perspectives on their professional responsibility for providing technical support to patients when completing the SPI using the app. Participants had a choice of in-person (i.e., ED site private office room), telephone or video-call interviews. The interviews lasted between 25 and 70 minutes and the first author conducted all interviews. Interviews were audio-recorded, transcribed verbatim, and anonymized prior to analysis. Prior to interviews, all participants signed an informed consent form after having received written information to enable them to make an informed choice regarding participation. The consent form was completed using REDCap following our organization standard.

### Data analysis

We analyzed interview transcripts using a directed content analysis [46], following the established guideline to use TDF and COM-B as a codebook [37] in NVivo 12. First, four reviewers (HDS, KD, IK, SK) read and independently coded four transcripts as a calibration exercise. Then, each of the remaining transcripts were independently coded by two reviewers. Reviewers and first author met to discuss and resolve any interpretation discrepancies. Once all codes were compared for consistency, we then generated participants’ specific beliefs within the TDF domains and indicated whether it is a barrier, facilitator, or mixture of both for influencing the target behaviour – using the app to complete SPI in the ED prior to discharging patients to home. Representative quotations from the interviews were selected to illustrate that findings are drawn from the participants’ original data. Data analysis involved 2 reviewers per each transcript to ensure that findings are the result of the experiences and ideas of the participants shared during the interviews rather than the characteristics and preferences of the lead investigator.

### Research team reflexivity

The first author (HDS), who led the data analysis, is a Registered Nurse and an immigrant of East Asian descent pursuing a PhD in Health Services Research. She has clinical experience and knowledge in emergency nursing, as well as extensive experience in qualitative research. Her clinical background and familiarity with the emergency care may have shaped her interpretations of the data. The additional reviewers (KD, IK, SK) on the team include master’s-prepared nurses and health informatics researchers with extensive qualitative research experience in mental health. The analysis was guided by COM-B and TDF, which provided a structured lens for interpreting the data while remaining attentive to interpretations that extended beyond the framework. To support reflexivity, the team engaged in ongoing discussions of their assumptions and emerging interpretations throughout the analytic process, considered alternative interpretations, and discussed the selection of illustrative quotes.

### Ethics statement

The research complies with all the relevant national regulations and institutional policies. Ethics approval for the study was obtained from the Research Ethics Board at the Centre for Addiction and Mental Health (REB# 2023/078) and the University of Toronto (REB # 45110). All participants signed an informed consent (written) form after having received written information to enable them to make an informed choice regarding participation.

## Results

A total of 29 ED clinicians were interviewed, including nurses (n = 14), psychiatrists (n = 7), social workers (n = 2), program assistants (n = 5), and a pharmacist (n = 1). Nearly half of these clinicians (n = 14) had between five and 20 years of clinical experience, while the remaining half (n = 15) had less than five years of experience. Seven interviewed participants had used the app, either with patients or without, but this was not exclusively in the ED setting because some staff cover shifts in different units within the same mental health hospital. A few additional individuals said they have heard about the app, but have not used it, while the remaining, more than half of the sample, were learning about the app for the first time during the interview. Participants identified themselves as men (n = 11), and women (n = 17). See Table 1 for participant characteristics.

**Table 1:** Characteristics of N=29 clinicians.

Characteristics	N	%
Clinical role		
Nurse	14	48.3
Psychiatrist	7	24.1
Program Assistant	5	17.2
Social Worker	2	6.9
Pharmacist	1	3.5
Gender		
Man	11	37.9
Woman	17	58.6
Questioning	1	3.5
Age		
<30	13	44.8
30-39	9	31.0
40-49	5	17.2
50-59	1	3.5
Racial background*		
White	14	48.3
Racialized (examples shown below)	16	55.2
East Asian	7	24.1
South Asian	5	17.2
Other categories include Southeast Asian, Black, Latin American, Middle Eastern		
Prefer not to answer	1	3.4
Years of experience in the current professional role		
<5	15	51.7
6-10	6	20.7
11-15	7	24.1
16-20	1	3.5
Current working hours		
Full-time	20	69.0
Part-time	8	27.6
Casual	1	3.4
Years of experience working in the current ED		
<5	19	65.5
6-10	5	17.2
11-15	5	17.2
*Non-mutually exclusive category		

Table 2 summary and the following section describe how the data maps onto the COM-B model and TDF, and a comprehensive list of barriers and facilitators along with additional quotes can be found in S3 Table.

**Table 2:** Summary of barriers and facilitators to using the app to complete SPI in the ED.

Domain	TDF	Theme	Subtheme	Barrier (B) / Facilitator (F)/ Mixed (M)	Example Quotes
Capability	Knowledge	Limited awareness of the Hope app*		B	“I don’t know much about them. I think this is the first I’ve actually ever heard of an app used for safety planning, like the type of safety planning that we use in the emerg. " [012]
	Knowledge and skills	Demonstrated knowledge and skills for safety planning using the paper version		F	“Safety planning, the purpose of that used in the emergency is to understand the client and their individual needs and what we can do to prevent further safety risks. That allows the clients to be more involved in their own care as well.” [004]
	Memory attention and decision making	Decision making and prioritization of tasks including safety planning		M	“It’s like a tool, right, it’s like a toolbox. You open your toolbox and use a special tool for whatever you need, right. It’s not a universal obligation for you. As a professional you figure OK, for this individual, this might work for this individual who’s probably not be able to access this app or access email if somebody, some person lives in a shelter, who has no phone, has no laptop or tablet, maybe has no email address either, probably paper copy will be better to keep on them. But majority of people probably should be able to utilise this app.” [002]
		Remembering the app especially in the beginning phase of change		B	“I think, to be honest, we see so many random posters and signs everywhere, it’s easy to just kind of tune them out.” [003]
	Behavioural regulation	Opting for the quickest way, and breaking habit		B	“I can see how, if staff is busy, if you have eight patients you need to run between, it’s easier to hand the patient here, a paper form and say, “Here, do it” rather than a patient be coming back to you, “How do I do this? How do I download it? My Wi-Fi is not working.” Yeah, I can see how it can be a barrier, but with technology improving, maybe Wi-Fi gets more stable here. Because quite often, they ask you for a password and all that stuff and password expires.” [002]
Motivation	Belief about capabilities	Level of technical confidence in using the Hope app		F	“I don’t have any problem with the technology of it. I think if I were to put in the time and the effort, then absolutely, it could work.” [009]
	Belief about consequences*	Perceived level of workload changes and difficulty in using the Hope app		M	“No, I don’t think that’s a huge workflow increase, no. Workload increase, no.” [007]“I think it might create new workload, in the sense of you might have people have, staff having to help patients figure out, how to download the app, how to open the app. And I think staff might see it as a more time consuming task.” [009]
		Uncertain benefits of safety planning beyond discharge		B	“It would be great to survey people who have actually downloaded the app, whether or not they find it useful. I don’t know. Because I never been in that sort of emotional predicament where I'm needing a safety plan for myself. So I don’t know whether it’s useful. I mean, objectively, it looks like it should be useful. But I don’t know whether we have feedback from end users” [007]
		Perceived benefit and disadvantages of the Hope app		M	“I think the other thing is, as I mentioned, having those toggles of just, oh, yes, this applies to me, this applies to me, might make it a little bit more passive.” [009]” I feel the app is really great, because it’s just easier to have in one place, especially when they're on their phone, and they can send it directly to whoever right away. And sometimes it’s just helps. It’s more than that. It’s better than writing because sometimes they can get so stressed out that they get fed up and they don’t want to write something” [010]
		Anticipation of patients’ reactions for checking the phone (Reading over patient’s phone may not be well-received)		B	“I think it makes it a little bit difficult in the sense that I'd have to go back in and see the patient and read their phone. Whereas in this scenario, it’s usually nursing brings me back the worksheet that the patient has completed. And then I just review it, copy it, give the original back to nursing so they can give back to the patient or give back to the patient myself.” [009]
		Beliefs about equity with regard to the Hope app		M	" I mean, the phone helps for younger folks or people that are interested in technology. But that’s not the majority, right, unfortunately. Well yeah, we see a lot of homeless people, impoverished people come in with police, they don’t have cell phones.” [005]“I think using cellphone for the population out there is feasible just because smartphone are so – they are everywhere.” [020]
	Goal	Safety planning is high priority for ED care		F	“Safety planning in the emergency department. It is always at kind of like top” [018]“Usually it’s a requirement for somebody that has depression or suicide. For some sort of history of self harming. Then for sure that they require, a complete safety plan, they can’t even be discharged. So, can’t really think of instances that I've seen where it’s been absolutely missed” [028]
	Professional responsibility*	Professional responsibility for safety planning		F	“I think it’s very important. Like it’s one of our – it should be one of our key assessments that we do as nurses here. Because even though the doctor, you know, is mainly the one who makes the decision about a disposition for a patient, our value as nurses is most of the time like taken into account by the doctors.” [012]
			Medical legal	M	“I don’t know if I if they did the hope app. I probably would just say they complete the hope app. And I eyeballed it looks good. And then if there was an adverse outcome, that’s all the information we would have, but I think that would be enough for the safety committee. And they would know they could pull up what is the Hope app? The recent version was that look like what would the client have filled? I think that would have been enough medical legally.” [027]
			Shared responsibility to support the Hope app implementation including tech support	F	“[Tech support is] something that we can all do. Because if we are familiar with the app, and we know how to use it, we should be OK” [010]“we also have other people that can help with some of those things like PAs can help with, with setting up the Internet, they can help them to open the app. And then if they need any more help, they can ask the nurses, so it’s not solely on the nursing staff.” [013]
			Variability in how clinicians approach safety planning related to time spent with patients	M	“Yeah, there are definitely certain days where there’s no time, but I think you have to make time. Especially for these vulnerable patients, right. And for me, personally, my practice, I don’t – especially these patients that are going through depressive episodes or have suicidal ideation, I never want them to think that I kind of abandon them. So for – the safety planning takes 10 minutes of your time, right, maybe a little bit more. But I don’t know, I think it's, I think this is a nurse to nurse question. And everyone’s practice is different. But I personally, even if I'm really, really busy, I will try to make time for these people.” [014]
		SPI is important professional role for all disciplines in the ED		F	“Safety planning is really important, right? We don’t let people leave hospital if they're not really to safety plan. And as a nurse, especially as a mental health nurse, I feel it’s my due diligence to safety plan, and the Hope app is something that they'll always have with them.” [014]
	Optimism/ pessimism	Personal preference towards digitizing healthcare services including safety planning		M	“If it’s not integrated with the health record, we would prefer paper because paper gets stand in.” [015]“I'm getting kind of tired with technology. And so maybe like what resistance do I have as a, I think my biggest resistance is probably paper to me feels a bit more therapeutic than a computer screen. Also, given the documentation doesn’t get into the charts. So it doesn’t feel as it might be more worthwhile for the patient. But from a clinician perspective, having a in the chart to me is very important.” [027]
Opportunity	Social opportunity	Communication for safety planning		B	“Every time a safety plan needs to be done the doctor has told me, “Can you just make sure they fill out a safety plan before they leave?” Other than that time I think I – well I guess it would be based on the nurse’s judgement whether or not the safety plan should be filled out. That’s actually a grey area for me. I'm actually not too sure if it’s every time the doctor says to do one, or if it’s based on also if we think that the patient needs to do one.” [025]
		Team culture in the ED		F	“Communication is important. And our team does that well. And we support each other outside of our roles, because we're family, so. And we try to pass that on to the patients for sure. And once we acclimate ourselves, I don’t see any problems rolling it out.” [005]
	Physical opportunity	Lack of access to smart devices among subgroup of ED patient population		B	“I think a lot of our clients who are homeless, living in shelter, they're chronically getting their items stolen because of the culture in shelters so many of our clients might get one phone and it’s gone in a week. Or with people with chronic addiction, they're selling their phones. " [012]
		Busy ED setting and being understaffed, need for prioritizing multiple tasks		B	“And I feel like it’s something that shouldn’t be rushed but it ends up being rushed because we – like, even today we have, like, five, six discharges happening at the same time. And if that’s the only thing that’s holding them back then, like, hey, as long as we have something on the paper then, like, you can go.” [016]
		Lack of documentation infrastructure in the EHR for the Hope app		B	“I think tools like a pop up reminder or a tab can be added to our multidisciplinary assessment.” [011]“But if it does become placed into the multidisciplinary assessment, which is the tool that we do for every emerge visit, if it’s part of that standard checklist of things to do, then it’s part of what we have to do so then it’s not, it won’t be overlooked.” [012]
		Wi-Fi and other technical resources in the ED		B	“I think the most challenging part is getting access to the Wi-Fi, because it’s like a long code to type. And then some patients, they can’t see properly so then we’ll have to take their phones and then we’ll type it in for them. " [023]“Yes, like I said, so if the patient was really eager to get discharged, telling they can stay safe. But I want to see a safety plan before they go. But now they're telling me, “Well, I can’t seem to download it. The internet’s really spotty.” Then we're kind of at, like, a difficult situation where I technically, I don’t have grounds to keep them. I can’t really imprison them in the hospital to do this app. To finish this.”[026]
		Organization culture – Alignment of the Hope app’s purpose with the organizational value and goal		F	“I think it fits with the organisation in the sense that [the organization] is continually striving to innovate the way that care is provided. So as I mentioned, it makes sense as generations change and things become more digital.” [009]
		App-specific	Layout, usability, resources,	F	“It’s pretty straightforward, I think that’s what matters. And like I said before, it's, multiple choice is always easier than to come up with something, less time consuming” [002]
			Lack of translation function in the app	B	“The multi language could be useful. Because that eliminates the needs of getting it translated and all that stuff.” [020]
			Toggle options in the safety plan	M	“The one other thing that I was just thinking of, I like that you have the little toggles to decide, if that applies to them, and then at the end, it’s optional to write in. Actually kind of feel like it’s sometimes beneficial that on the worksheet, it’s totally blank. And they have to think and write it in. Whereas they feel like there might be a passive kind of just, yeah, sure, sure, like checking off a bunch of ones. And it’s hard to tell then if the safety planning was really thought through.” [009]
	Social opportunity	Leadership support for change management		F	“If [leadership team] asks us to implement something in our practice, I think everyone is pretty good about it.” [014]
		Patients’ readiness for safety planning		B	" And there’s been instances where they, for example, just say that there’s nothing. Like they can’t fill it out at all, they have nothing to write. And that becomes a little bit tricky to kind of persuade them. Especially when we're really rushed in the emerge and there’s so many other clients to be seen. It can be difficult to kind of engage them meaningfully.” [024]
		Patient characteristics in the ED (including age, preference, mental health conditions)		M	“. So, I mean, the phone helps for younger folks or people that are interested in technology. But that’s not the majority, right, unfortunately.” [005]“I got to say, even elderly people with cognitive impairment, if they have a smart phone, this is not a problematic app to use. It is a short download, people find it, they find it easy to use. If they have a smart phone.” [024]
		Alignment of the app’s purpose and ED and organizational goal/vision		F	“I think [the organization] is, is really great like that we were always sort of ahead of the curve, I think. And when people come here, they're always very impressed by all the technology and all the new things that we do. Everything like that. So that’s pretty cool.”[013]

* Salient themes.

### Capability

The COM-B defines psychological capability as the skills, knowledge, and ability to engage in the necessary reasoning and decision-making processes [34]. Related TDF domains under capability include knowledge, skills, memory, attention, decision making, and behavioural regulation, such as intentional habit breaking [36]. Clinician’s psychological capabilities influenced their use and non-use of the SPI app.

#### Limited awareness about the Hope app.

Most notably, clinicians’ reasons for not having used the app were primarily related to unawareness, even though the app had been available since 2020 and was developed by the organization. There were pilot implementation efforts and promotional activities including social media posts, internal email communications and internal web posts with the app’s launch, but no widespread or sustained initiatives have been undertaken across the organization leading to its implementation in routine workflows. It appears to have had limited penetration into the ED setting. Moreover, limited awareness could also have been potentially be attributable to staff turnover in the ED, especially during the pandemic; there were occasional new staff members, and residents rotated throughout the health institution. Even if clinicians had heard about it through organizational emails, they lacked knowledge about the app’s features and contents. Few clinicians knew that the app included wellness activities such as journaling and boxed breathing, as well as community resources tailored to users’ locations.

*"So what I know is, it’s a free phone app that anyone can download. And there’s some tools on it. Like suicide safety planning. And I believe there’s breathing exercises, grounding exercises, and perhaps some maybe quick phone numbers for the hotlines. That’s from my memory." [011, social worker]*

*"I don’t know much about them. I think this is the first I’ve actually ever heard of an app used for safety planning, like the type of safety planning that we use in the emerg." [012, nurse]*

*"I didn’t know anything about it until I read your email. But I all I know is that it’s related to creating safety planning for patients." [016, nurse]*

#### Remembering the app.

Clinicians mentioned that information (i.e., posters, flyers, etc.) about new innovations, like the app, often goes unnoticed due to the abundance of other posters. For this reason, clinicians expressed concerns that it may be difficult to remember to use the app to complete safety planning, especially during the early phase of implementation and when clinicians are managing multiple priorities simultaneously.

*“I think, to be honest, we see so many random posters and signs everywhere, it’s easy to just kind of tune them out.” [003, psychiatrist]*

#### Opting for the quickest way, and breaking habit.

Clinicians emphasized that in order to transition away from using paper SPI in the busy ED setting, the value proposition must be clear. In other words, the app implementation and use must be easy and more efficient than the current method of delivering safety planning. This also relates to both the physical environment and clinicians’ anticipation of future outcomes. Due to the nature of the busy ED setting, clinicians expressed that they tend to gravitate towards the quickest way to complete assigned tasks, including safety planning. Since the app will not replace the paper version entirely, this option will remain available. Thus, if instructing patients to download the app or providing technical support to patients proves to be time consuming, clinicians will likely opt for the quicker method, which is using paper.

*“I can see how, if staff is busy, if you have eight patients you need to run between, it’s easier to hand the patient here, a paper form and say, “Here, do it” rather than a patient be coming back to you, “How do I do this? How do I download it? My Wi-Fi is not working.” Yeah, I can see how it can be a barrier, but with technology improving, maybe Wi-Fi gets more stable here. Because quite often, they ask you for a password and all that stuff and password expires.” [002, nurse]*

#### Demonstrated knowledge and skills for safety planning.

Since safety planning is a standard discharge practice in the ED, the majority of interviewed clinicians demonstrated knowledge and skills for completing safety planning using the paper version. Often, nurses, social workers and psychiatrists are directly involved in safety planning. Program assistants, who are not directly involved in safety planning process, also seemed to have a good understanding of safety planning and its purpose.

*“What the warning signs are, coping strategies. What their support system looks like. How they can keep themselves safe, their reasons for eliving. What they could do when they're in crisis, and who they can reach out to when they're in crisis” [013, nurse]*

### Opportunity

Barriers and facilitators within the social and physical ED environment can shape the opportunities for clinicians to use the app to deliver SPI. Physical opportunity is determined by environmental factors such as time, location, and physical and human resources in the ED [36]. Social opportunity refers to social factors such as cultural and social norms, peer pressure, relationships, and social cues that influence behaviors [36].

#### Communication about safety planning in the ED amongst clinical team.

There is currently no standard method of communicating about safety planning in the ED amongst the clinical team. Safety planning is not included in order forms in the electronic health record (EHR). Moreover, participants described that safety plans can be initiated by psychiatrists, social workers, or nurses. When safety planning is initiated by a psychiatrist and completed by a nurse, communication primarily takes place verbally. Alternatively, nurses sometimes discovered that a safety planning paper was provided to the patient by a psychiatrist. This barrier was not specific to app-based safety planning but rather applied to safety planning in general, which may impact the adoption of the app and add an additional layer of socio-technical complexity in routine practice.

*"There’s suicide risk assessment and suicide evaluation. But there’s no checkbox for was a safety plan administered? Or should we do it? It’s just case by case? We feel in the moment that clinically it’s a necessary tool, then we'll, the doctor will say let’s do a safety plan or the social worker would say let’s do a safety plan. Then we do it and then review it." [012, nurse]*

*"In my experience, every time a safety plan needs to be done the doctor has told me, “Can you just make sure they fill out a safety plan before they leave?” Other than that time I think I – well I guess it would be based on the nurse’s judgement whether or whether or not the safety plan should be filled out. That’s actually a grey area for me. I'm actually not too sure if it’s every time the doctor says to do one, or if it’s based on also if we think that the patient needs to do one." [025, nurse]*

#### Lack of documentation infrastructure in the EHR for the app.

Another barrier was related to the current documentation infrastructure in the EHR. Currently, the patient’s paper safety plan is photocopied and scanned into the EHR. If the app-based safety plan were to be used, clinicians were uncertain about how they would document or keep record of the completed digital safety plan in the EHR. Clinicians also discussed the medical-legal responsibilities and the importance of documenting the safety plan; they said safety plans provide them with an idea of whether patients can be safely discharged to home and allows for the assessment of patients’ insights to manage their symptoms. As such, clinicians discussed the opportunity to change or add in a new form within the EHR to document completion of a safety plan.

*“One other issue that I'm just now thinking about is that, as I mentioned, we like having the safety plan to put in the file. We like having – before I discharged someone, I like seeing that they filled out the safety plan. And if it’s on their phone, I kind of wonder how I would be able to see that? Would I have to go and look on their phone in order to see that they filled things out? Because that sounds like it would be – it’s a lot easier then for the patient to say they'll do it, and then not fill it out.” [009, psychiatrist]*

#### Patient characteristics in the ED.

Patient characteristics tend to influence clinicians’ decisions regarding the use of the app for safety planning. Clinicians often mentioned that younger patients who visit the ED would benefit the most from the app due to their widespread use of smartphones and familiarity with apps. Conversely, clinicians assumed that older patients, particularly those who may not be as tech-savvy or reliant on smartphones, might face challenges in engaging with the app for safety planning. This assumption may influence clinicians’ decisions to offer the paper version rather than the app.

*" I got to say, even elderly people with cognitive impairment, if they have a smartphone, this is not a problematic app to use. It is a short download, people find it, they find it easy to use. If they have a smartphone." [024, program assistant]*

*"I think it depends on the age range. I found with younger clients that are in their teenage years or maybe a bit more reluctant to fill out a physical paper or early twenties they don’t want to do it. I'd rather scroll on their phone. So I think the app might actually be a solution to that. It'd be easier to kind of click through the questions and type it versus writing on a physical paper that they're going to lose right away." [028, nurse]*

#### Lack of access to smart devices among subgroup of ED patient population.

In addition to patient characteristics and preferences, clinicians also discussed physical access to devices for downloading the app. They mentioned that individuals experiencing homelessness tend to misplace their belongings, but this barrier applies to both modes of SPI. Additionally, clinicians noted that there are patients who do not have smart devices (i.e., flip phones) and will require a paper copy of the safety plan.

*"I think a lot of our clients who are homeless, living in shelter, they're chronically getting their items stolen because of the culture in shelters so many of our clients might get one phone and it’s gone in a week. Or with people with chronic addiction, they're selling their phones. " [012, nurse]*

*"I think the barrier, from my perspective, is that people need to have a smart device. Yeah. Or phone… I know that probably most people do now, but we also do work with people who are experiencing homelessness or poverty. So not everybody has access to that… like I said earlier, like people can have technological challenges, which again, we can help problem solve. But sometimes people just don’t like to use apps and that’s a preference thing" [029, psychiatrist]*

#### Wi-Fi and other technical resources in the ED.

Although the ED has public Wi-Fi for patients’ use, clinicians expressed concerns about its speed, noting this as a potential barrier for using the app. Additionally, during night hours, access to the ED Wi-Fi requires a password. The current process for accessing public Wi-Fi in the ED is not simple. Therefore, if patients awaiting discharge are delayed due to app downloads, this would not be ideal, especially considering the nature of the ED.

*“We shut [Wi-Fi] off at night and you need weird passwords, like it’s – I'm sure from a security standpoint, it’s quite adequate. But in terms of accessibility for patients that are stuck here, the internet is not very good here.” [005, program assistant]*

*“If the patient was really eager to get discharged, telling [me] they can stay safe. But I want to see a safety plan before they go. But now they're telling me, “Well, I can’t seem to download it. The internet’s really spotty.” Then we're kind of at, like, a difficult situation where I technically, I don’t have grounds to keep them. I can’t really imprison them in the hospital to do this app. To finish this.”[026, psychiatrist]*

Clinicians also discussed access to computers in the same building for public use, as well as available tablets in the ED. Since the app has an email function to share the safety plan, clinicians talked about alternative ways for patients to access a digital version of the safety plan, particularly for individuals who do not own smart devices but would still like to have a digital copy of the safety plan.

*“So some do have emails and the access to a computer can be sometimes arranged. They can even come here… …We can do it with them. Or we have some tablets. And we can do that with them so I can leave it with them to do here and email it to me and I could print it out for them. But then I’m not sure if that’s – depending on – it may be better than the one we have now because they can put their own options in and I think that would be the benefit of it.” [018, social worker]*

#### Busy ED setting and being understaffed, need for prioritizing multiple tasks.

The ED is busy most of the time, with clinicians having multiple priorities depending on patient acuity, turnover and staffing levels. While the busy nature of the ED was not an issue specific to the app, it may serve as a barrier due to the added layer of complexity if the implementation process does not streamline the existing procedures.

*"And I feel like [safety planning is] something that shouldn’t be rushed but it ends up being rushed because we – like, even today we have, five, six discharges happening at the same time. And if that’s the only thing that’s holding them back then, as long as we have something on the paper then, you can go." [016, nurse]*

*“All the little things add to each other. And in the ED, nurses have to be available for the patients, all of the patients they have in case of emergency because code whites and code blues can happen very frequently. So a nurse cannot stay with a patient for a long time to go do all this because they’re expected to do the initial assessment as well” [006, nurse]*

#### Team culture in the ED.

A strong culture of team-based interdisciplinary collaboration was present in the ED. This culture was a significant facilitating factor for implementing new initiatives, including the adoption of the app. Clinicians mentioned how technical tasks, such as assisting patients with accessing Wi-Fi, were often a shared task amongst the team.

*"Communication is important. And our team does that well. And we support each other outside of our roles, because we're family, so. And we try to pass that on to the patients for sure. And once we acclimate ourselves, I don’t see any problems rolling it out." [005, program assistant]*

*“I think it’ll take some change management, for sure. But I think the team is very strong and, like, very open to trying new things.” [015, nurse]*

*“I found the team is really cohesive in the emerge. It’s a really great team to work with”[024, psychiatrist]*

#### Leadership support for change management.

Clinicians spoke about leadership support and how they are aligned with the leadership’s decisions. Clinicians often mentioned that if the app adoption is made mandatory by the leadership team, it will be well accepted by the ED team.

*“I think the leadership should promote this behaviour because we are not asked to do this with the patient, so we just don’t do it.” [006, nurse]*

*"If [leadership team] asks us to implement something in our practice, I think everyone is pretty good about it." [014, nurse]*

*"I think if we can, you know, get people on board with it through leadership, like, endorsing it, through physician leadership endorsing it, some champions, maybe, or whatever we want to call them, then yeah, I think it could work well. But it will take time. Practice change takes a lot of time." [015, nurse]*

#### Alignment of the app’s purpose and ED and organizational goal and vision.

Clinicians unanimously spoke about the alignment of the app’s purpose with the organizational vision. As Canada’s largest academic mental health hospital, clinicians viewed the organization’s vision as leading innovation and staying ahead of innovative trends, including the use of apps.

*“I think [the organization] is really great like that we were always sort of ahead of the curve, I think. And when people come here, they're always very impressed by all the technology and all the new things that we do. Everything like that. So that’s pretty cool.” [013, nurse]*

### Motivation

Motivation encompasses the brain processes that guide our decisions and behaviors [36]. The COM-B distinguishes between automatic, such as emotions and impulses, and reflective motivations, including beliefs, values, evaluations, and plans for a target behavior [34]. Several barriers and facilitators for using the app for safety planning were found to be associated with clinicians’ motivations.

#### Professional role and identity related to safety planning.

The interviewed clinicians unanimously emphasized SPI as a priority care goal in the ED, closely connected to their professional role and identity in ensuring patients’ safety. Safety planning is already integrated into standard discharge practice and is considered fundamental to the care provided in the ED, described as the “bread and butter” of their work. For such reasons, the introduction of a new modality to improve the portability of safety plans was mostly well-received by clinicians.

*“Safety planning in general. I mean, I think that that’s part of our assessments, that’s really the sort of bread and butter of what we do in the emergency department. And safety assessments to some degree, are sort of incorporated into every assessment and discharge that we do… … I think there’s a variety, I think there’s sort of a spectrum of different safety assessments, as I said, depending on the patient, the provider, the risk threshold, all of these things. But I do think they're incorporated into most if not all of the assessments.” [007, psychiatrist]*

*“I almost feel obligated to – like, it’s not my responsibility to keep people safe at all times. But at least while I’m working here, like an ED, I feel like anyone that comes to seek for help, I feel obligated to do my best to keep them safe. And just because they come at the lowest moment of their life and, I’m able to help – that’s great. But if I don’t do enough then, I question myself, you could have done better. You could have spent more time with that person. Or just because I was preoccupied with something else and I couldn’t spend more time with that person, that person could have gotten better care. So I feel like safety planning is part of me doing my obligation in by sitting with them and just go through the questions with them.” [016, nurse]*

Despite the importance of safety planning in the ED, variability in its delivery has been identified. Although crucial for all clinicians, the level of interaction provided to patients regarding safety plans differed. Clinicians discussed that there is currently no prescribed time that clinicians need to spend on safety planning with their patients. Rather, it is based on the clinician’s discretion, whether the patient requires extra support when completing the safety plan for the first time or is already familiar with it. Therefore, when considering the app version of the safety plan, the amount of time that clinicians felt they need to allocate to each patient varied. Although this was related to the busy nature of the ED, clinicians viewed safety planning as an important role regardless of the time allocated to this task.

*“Yeah, there are definitely certain days where there’s no time, but I think you have to make time. Especially for these vulnerable patients, right. And for me, personally, my practice, I don’t – especially these patients that are going through depressive episodes or have suicidal ideation, I never want them to think that I kind of abandon them. So for – the safety planning takes 10 minutes of your time, right, maybe a little bit more. But I don’t know…I think this is a nurse to nurse question. And everyone’s practice is different. But I personally, even if I'm really, really busy, I will try to make time for these people.” [014, nurse]*

Considering the new modality, clinicians discussed how they saw technical support as part of their role. Technical support includes, but not limited to, assisting patients with Wi-Fi connection, downloading the app, or orienting patients with the app features. Although these tasks are not traditional ‘clinical’ roles, clinicians discussed that they have been providing such support to patients already in the ED due to the ubiquitous use of technologies in healthcare. However, they talked about how important it was to share this responsibility among the team.

*"[Tech support is] something that we can all do. Because if we are familiar with the app, and we know how to use it, we should be OK" [010, program assistant]*

*"We also have other people that can help with some of those things like [program assistants] can help with, with setting up the Internet, they can help them to open the app. And then if they need any more help, they can ask the nurses, so it’s not solely on the nursing staff." [013, nurse]*

#### Perceived benefits and disadvantages of the app.

Clinicians discussed anticipated benefits of the app. Some talked about some patients currently leaving the ED without their paper safety plan, so the portability aspect of carrying it with them via a phone was highly valued.

*“The paper you can leave in your car, in your home, in your bag and or lose it and eventually – with the app, you can always open it up and then remind yourself it’s always with you, if you want it, instead of trying, OK, I left my safety plan at home. But now at my parents’ house, I don’t feel safe, I feel distressed and tried to remember what I did. And this way I have it on you. I think that’s a big advantage." [002, nurse]*

*"I do support going paperless, it’s just tough, I think. Then also patients can easily lose that, there is a benefit to the app in the sense that those who have phones have their phones with them all the time." [009, psychiatrist]*

*"I think it’s so great that they can carry it with them when they leave. Because people –especially people, they don’t care about paper like that. They'll throw it out, but on their phone, they can take it everywhere… When we're cleaning the rooms, we see so many safety plans left behind." [010, program assistant]*

A few clinicians commented on the layout of the safety plan in the app, suggesting that it prevents genuine reflection compared to the paper copy of the safety plan, which requires patients to come up with their own answers. Conversely, some mentioned that the app is more helpful and reduces time because it offers prompts to stimulate patients’ thinking.

*“I just like to know that they've really thought about what responsibility they have in their own safety and what resources are available to them. It’s kind of passive versus active learning. Like in the process of them writing out what resources are available to them, they're kind of actively participating in their own safety.” [009, psychiatrist]*

*“I'd say list the strategies and as you come up with those strategies, and sometimes you have to sort of brainstorm with the patients, or often solutions tailored to them. Because if you say to one individual that, “Well, you do this”, “Well, I don’t have access to my family, I'm alone in this universe” type of thing. So it can be triggering to some people. But if it’s just a select few options, that'll probably limit that, so it’ll be easier, you just tick, tick, tick.”[002, nurse]*

#### Perceived impact of safety planning.

There were discussions regarding the anticipated impact of safety plans in general. As SRTB exists on a spectrum, patients’ engagement with their personalized safety plans to manage their daily lives extends beyond the control of the ED team. Relatedly, there appeared to be some disbelief or skepticism towards the effectiveness of safety plans despite the existing evidence. Such beliefs need to be addressed, as they can affect the existing SPI practice in the ED and further challenge the adoption of the Hope app.

*"I think it’s useful, more mostly in the clinician’s perspective, as opposed to the patient’s perspective. And that’s only because, like I mentioned that piece of paper, once we've given it to them, more times than not, they just throw it out, it doesn’t actually mean too much to them. I think out of 10 patients, maybe three or four would actually use that as the set purpose, the safety plan." [003, psychiatrist]*

*"I will say the biggest thing is patients really like it. And like I said, a lot more than they like the paper. Not that they ever minded the paper, but they really like it, and they really like having it on their phone and being able to open it easily and it feels like, I think, like this comforting thing that you can’t lose the way that you would immediately lose the paper. And that it’s a working document and that you can change it" [024, psychiatrist]*

*“I mean, they'll always be the person that thinks that it’s rubbish. I mean, patient wise, right. Where they're just like, this isn’t going to do anything. I'm going to scribble a couple answers that you like, down on a piece of paper, and you're going to discharge me now. It’s like a check mark on the chart. But then there are some people that it’s a good point of reflection, and then they rely on those new coping mechanisms when they're struggling in the future.” [005, program assistant]*

#### Perceived level of workload changes and difficulty in using the app.

Clinicians discussed workload changes regarding the implementation of the app. Some believed the app modality would decrease workload by minimizing time to retrieve and scan the paper forms. On the other hand, some were concerned that the app would increase their workload due to providing technical support to patients, potentially delaying discharge, given current Wi-Fi limitations in the department (opportunity). Uncertainty regarding workload changes was an important consideration to address for future implementation efforts.

*"I think it might create new workload, in the sense of you might have people, staff having to help patients figure out, how to download the app, how to open the app. And I think staff might see it as a more time consuming task." [009, psychiatrist]*

*“I just think that it would be kind of a little bit time consuming. Of course, we don’t mind, but it’s just that there’s not always going to be that chance to – it might not be even often, but you know, but it would all come down to how the day is going and how busy is that day.” [025, nurse]*

*"I think the practice change is going to be relatively easier. Because from my perspective, it just comes across, like I said, of having a tool on hand in my pocket, that will actually help me explain a safety plan better. And then I still have the option of offering the paper copy, which I generally offer anyways. The only thing is it requires more time." [003, psychiatrist]*

*"I think we are technology savvy enough for each team member to if they need to sit down and show the patient how to use it. But that also takes time and that’s the thing that’s in short demand in the [ED]. So, everybody is just so busy. So initially, just kind of sitting down and going through with patients how to use it, I can imagine staff members will feel like that’s another burden on their time." [017, pharmacist]*

#### *Anticipation of patients*' *reactions to checking the phone.*

Another barrier discussed was concerns about patient’s reactions to clinician’s viewing the safety plan on the patient’s personal device. Although it is the clinicians’ responsibility to support patients in developing and going over the safety plan, they were unsure whether it would be appropriate to ask patients to hand over their phone or turn the screen towards clinicians. In either case, clinicians were concerned about how patients would react. Some mentioned that it would be ‘invasive,’ potentially impacting the therapeutic relationship.

*"I think it makes it a little bit difficult in the sense that I'd have to go back in and see the patient and read their phone. Whereas in this scenario, it’s usually nursing brings me back the worksheet that the patient has completed. And then I just review it, copy it, give the original back to nursing so they can give back to the patient or give back to the patient myself." [009, psychiatrist]*

*"Looking over somebody’s phone is invasive a little bit? Yeah, a little bit. And not everyone might be comfortable to do that or to hold the phone itself or with COVID germs." [012, nurse]*

## Discussion

Grounded in behavior change theory, the COM-B and TDF facilitated a comprehensive analysis of clinicians’ capability, opportunity, and motivation for app use, shedding light on how these factors interact to influence behavior. We had interviews with 29 ED clinicians, comprising nurses, psychiatrists, social workers, program assistants, and a pharmacist, with varying levels of clinical experience. Most participants had not used the app, highlighting a lack of awareness despite its availability since 2020. Clinicians mentioned staff turnover rate, busy schedules, and an overflow of information as contributing factors to their limited awareness for the app. The study revealed key barriers such as the absence of standard communication methods for safety planning, limitations in EHR documentation infrastructure, and concerns regarding patient access to smart devices and Wi-Fi connectivity in the ED. In addition, clinicians discussed the anticipated consequences of workload changes in an already busy ED setting. Despite the perceived barriers, clinicians expressed positivity to adopt the app, emphasizing its potential benefits in enhancing safety planning portability and patient engagement. Notably, strong team culture, leadership support, and alignment with organizational goals emerged as facilitators for the app use in routine care. These findings underscore the need for tailored strategies addressing clinicians’ capabilities, opportunities, and motivations to promote successful implementation of the app in the routine clinical care.

An important assumption about human behaviour is that barriers and facilitators within the COM-B and TDF domains are not static but can interact with each other to influence behaviour change [35]. In other words, individuals’ capability influenced aspects of their motivation, like the level of confidence, and factors related to opportunity, like the social environment or team culture, also influenced motivation to perform the target behavior. For example, physical resources such as slow Wi-Fi in the ED influenced clinicians’ beliefs about anticipated changes in workload, creating a negative emotional response, which influenced the likelihood of performing the target behavior – using the app to deliver SPI. As such, when designing implementation strategies, it is important to think comprehensively about the possible behaviour change solutions to target multiple COM-B domains [35].

One major gap in implementation science is the absence of theoretical grounding in strategy development, alongside a mismatch between context and implementation strategies. Such a gap often leads to incomplete and unsustainable implementation [47]. To understand how the barriers can be addressed and facilitators can be leveraged, we examined suggested intervention functions (i.e., solutions) for the target determinants of behavioral change. Table 3 has been informed by the BCW, work of Michie et al. [35], and displays the matrix of intervention functions deemed most relevant to target the COM-B barriers, colored in green. These connections between intervention functions and COM-B domains have been previously established through expert consensus and reliability testing [35]. We mapped our study findings to the left column according to the COM-B. By using this matrix, we identified appropriate types of implementation strategies that target the barriers and leverage facilitators to support clinicians’ behavior change.

**Table 3:** COM-B matrix and intervention functions.

Barriers and facilitators identified from the current study	COM-B	BCW Intervention Functions								
		Education	Persuasion	Incentivization	Coercion	Training	Restriction	Environmental Restructuring	Modelling	Enablement
Awareness, knowledge, skills, memory attention, decision making processes, behavioural regulation and habit breaking	Psychological capability									
Wi-Fi and technical resources, documentation infrastructure, access to smart devices, busy ED, short staffing, patient population	Physical opportunity									
Communication about SPI, team culture, leadership support, organizational vision	Social opportunity									
Professional role and identify, preferences for technology, technical competence	Autonomic motivation									
Professional role and identify, beliefs about consequences for using the app, perceived impact of SPI, goal	Reflective motivation									

The matrix confirms that to address limited awareness of the app, education and training is necessary to support the implementation. Web-based training and informational resources about the app are available within the organization, but these educational tools may require updating and active dissemination efforts in the ED. Training and education are popular implementation strategies for suicide prevention [26,48]; however, it is important to note that addressing limited awareness and promoting familiarization of the app alone cannot lead to successful implementation. There are other barriers we identified that require more attention, such as motivation and opportunity, which are often not adequately addressed and lead to ineffective implementation in ED related to suicide prevention care [49]. To address concerns related to the busy ED and workload challenges, environmental restructuring may be necessary. Environmental restructuring refers to making changes in social or physical environment [35]. For example, leadership commitment, as emphasized in the Zero Suicide framework [50,51], may help establish organizational expectations that further support, elevate, and/or invest in suicide prevention efforts, including SPI. Other ideas include making EHR form changes to streamline the SPI documentation process and standardizing communication process that clinicians are concerned about. However, these changes related to documentation and communication processes within the ED are not simple. For example, considerations include where SPI delivery should be documented within the EHR and how its initiation and completion should be communicated across the ED care pathway. These changes will require close collaboration with clinicians to determine what solutions are feasible and sustainable. Additionally, to address skepticism towards the effectiveness of SPI, persuasion may be necessary. Persuasion-type interventions can be particularly helpful in mitigating negative emotions or attitudes [35]. For example, referring staff to systematic reviews on the effectiveness of SPI and patient testimonials for the app and its impact on their SRTB management can help increase their awareness of the positive impacts that SPIs can have on suicide prevention.

This mapping exercise provided insights into the various combinations of intervention functions needed to target specific COM-B determinants of behavior change identified in this study. While intervention functions are broad categories of techniques, behavior change techniques (BCTs) –the smallest units (or more specific components) of implementation strategies [52] – will be selected in the next phase in collaboration with clinicians, patient and family partners. There are 93 possible BCTs linked to intervention functions [35,52]. Corresponding BCTs for each intervention function, or “active ingredients” for changing behavior, will ensure that the implementation strategies are theoretically grounded and tailored to achieve the desired behavior change. The findings from this study laid the foundation for the subsequent design process, which aligns with evidence-based practices for creating rigorous implementation strategies to make the tailoring process more effective [53].

Anticipated concern for workload changes associated with the app implementation was one major concern that we heard from many clinicians. This finding is corroborated by a previous qualitative systematic review, which suggests that the integration of new technology can be perceived as creating an additional burden of care, including administrative tasks related to technology [31,54]. Lack of time and additional tasks for a newly introduced technology was also discussed, similar to the current study findings. Additionally, limited infrastructures for supporting new technology implementation in clinical settings [31] were also similarly identified in the current study. Integrating apps into the EHR is one effective way to support sustainable integration of these interventions [55]. Linking the app to EHR systems can positively impact health care delivery, as EHRs are already part of clinicians’ workflows, and this integration has been shown to support clinical decision-making by enabling patient-entered data readily available to clinicians [56]. As such, various interoperability standards exist to support the linkage of apps with the EHR, such as the Health Level 7 Fast Healthcare Interoperability Resources (HL7 FHIR) [57]. However, this is not always feasible within the healthcare organization, especially as FHIR itself faces its own implementation challenges [57].

We also identified several facilitators, which represent strengths that can be leveraged for future implementation efforts for the app. Notably, within this ED, we observed a strong sense of professional responsibility, a cohesive team culture, and strong leadership support. While targeted implementation strategies are often customized to tackle identified barriers, as recommended in implementation practice, it is also equally important to capitalize on existing facilitators when supporting implementation. One critical facilitator for technology implementation in healthcare is leadership and team culture [26,54,58]. The current study setting is in an advantageous position due the robust leadership support and positive team dynamics. While the lack of leadership support and the absence of positive team dynamics have been barriers in previous mental health app implementations [59], in this setting, these factors can be capitalized as strengths to sustain the app implementation and adoption. Despite this strength, it is also essential to consider the non-static and iterative nature of healthcare systems. Sustainability (i.e., long-term use and maintaining benefits) remains to be one major gap for implementation of evidence-based interventions in healthcare [60–62]. Team culture and leadership may evolve over time, and new barriers may arise during the implementation process. Therefore, while we can capitalize on and sustain these existing strengths, it is imperative that we continue to monitor them. This includes making necessary adaptations [63] to the deployed implementation strategies and considering the introduction of new strategies [61] to ensure the sustainability of the app use for SPI.

As more innovative technologies are integrated to augment care, clinicians are increasingly assuming non-traditional clinical roles, such as supporting patients in connecting to Wi-Fi and troubleshooting technical glitches. Although these roles are not traditionally considered clinical, clinicians in the current study reported that they undertake these tasks, nonetheless. This was identified as a facilitator in the current study; clinicians were already providing this technical support to patients in the ED, primarily with Wi-Fi connection. Additionally, there may be added tasks with the app implementation, such as introducing and orienting the app to patients. To ensure the sustainability of the app implementation, further exploration is necessary, as technical support cannot always be guaranteed due to human resource challenges and increasing patient volumes [64], potentially limiting clinicians’ availability to provide such support to patients. Digital navigators are an emerging area to support the adoption and integration of technologies into care [65,66]. Major roles of digital navigators include technical assistance [65], workflow optimization [67,68], and providing support for digital literacy [69], among others. Digital navigators have also been implemented to achieve digital healthcare equity, as they can help individuals with low digital literacy to meaningfully use digital technologies to achieve desired outcomes [69]. Similarly, in the current study, program assistants were more frequently involved in tasks resembling those of digital navigators compared to other clinical disciplines. To ensure the sustainable implementation of technologies in healthcare, formalizing digital navigator roles could offer potential solutions to address human resource challenges.

Clinicians were concerned about potentially infringing upon their patient’s privacy by having to view their patients’ personal device, despite clinical responsibility for assessing the completeness of the safety plan. As SPI is inherently collaborative, even with changes in modality, it is expected to maintain its collaborative nature. As such, the use of apps and other digital tools in healthcare raises questions about how it can be therapeutic and collaborative [70,71]. Additionally, SPI is one critical aspect of discharge planning. Discharge communication can be seen as an essential transition point between hospital care and at-home recovery and management. Discharge communication and instructions delivered during this time are crucial in all healthcare settings, including the ED. Despite this, ED clinicians face unique challenges due to the busy environment. For instance, the discharge process may be rushed, which can contribute to poor discharge communication [72], leading to insufficient instructions [73], impacting patients’ post-discharge health outcomes and increasing return visits to the ED [74]. Considering the nature of SRTB and the critical window of opportunity for healthcare contact by patients [75–77], the discharge process must not be rushed. Further exploration is needed to determine how the app can be integrated into clinical workflows without negatively impacting clinicians’ workload. In particular, there is a specific need to determine how the app can be introduced in the ED to maintain the collaborative nature of SPI, as well as when, in relation to discharge, it should be introduced during the patient’s stay in the ED. As outlined in the next phase of the study [41], such details will be explored and discussed with a team of patient partners, families, and clinicians to co-develop implementation strategies.

Prior implementation studies of SPI apps in European countries have highlighted limited uptake and sustained use of these apps over time, despite their high ratings for usability and acceptability [78]. Recent research in New Zealand has also shown a positive attitude towards digital tools among mental health clinicians, with smartphone apps being frequently utilized alongside other types of digital tools [79]. Similarly, clinicians in the current study expressed openness to the app because of its ease of use and their perceived digital literacy skills. Our study explored factors beyond usability and acceptability (i.e., motivation, opportunity) and their relationships, shedding light on potential explanations for the limited uptake and sustained use, and highlighting the importance of considering the dynamic nature of the healthcare system and the individuals involved when implementing new innovations. Additionally, previous research has used the BCW and TDF to investigate behavior change strategies aimed at encouraging primary clinicians to prescribe apps [80]. Similar to our findings, education and training were identified as necessary strategies to support clinicians in improving knowledge, self-confidence, and skills for using apps. However, none of the behavior change strategies identified in the current literature used rules or restrictions to increase app prescriptions or decrease competing behaviors [80]. In line with recommendations for behavior change solutions [80] and the findings presented in Table 3, our future work will involve co-design sessions with clinicians, families, and ED service users to identify feasible behavioural strategies and ensure that their format and delivery are appropriate and accessible to busy ED clinicians [81]. We will also maintain the theoretical backbone during the future design process by selecting effective BCTs within the identified intervention functions of the current study [41]. This approach will ensure that the implementation strategies are theoretically grounded and effectively tailored to achieve the desired target behavior change.

### Limitations

This study’s findings are limited to one ED context, which is unique, compared to other EDs that provide both medical and psychiatric emergency care services. As such, findings may have limited transferability, and there may be additional barriers unexplored due to the nature of the study context. For example, EDs are often described as having a biomedical focus, leading to hesitancy and uncertainty from the ED team towards psychiatric care [82]. In general, the processes for managing psychiatric conditions in the EDs are not as well developed as for managing physical conditions. While the ED has established procedures for transferring or admitting patients with acute physical conditions to specialized care after medical stabilization, a similar level of confidence and system support for mental health cases is lacking [83–85]. As such, the current literature highlights the need for improved training support for ED staff in psychiatry with a growing recognition that the ED often serves as a point of entry for individuals with SRTB [86–88]. Such a gap in ED care is attributable to the existing dichotomy of care between psychiatry and medical/physical health care, which needs to be further challenged to promote holistic healthcare [82]. However, since the ED in this study provides psychiatric emergency health care exclusively, this could have led to a different culture, different attitudes and levels of professional role and identity related to SPI. Also, as a psychiatric ED, it has a high level of mental health staff resources which may not be available in other ED settings. To accommodate for these contextual differences and to support transferability of our study findings, we have attempted to provide contextual details where appropriate. Future exploration of ED culture and professional role identity associated with suicide prevention care among clinicians in different ED settings may be beneficial for identifying nuanced contextual differences. Such findings could help shape strategies to influence a culture that promotes a strong sense of responsibility toward suicide prevention care and instills the belief in the interconnectedness of physical and mental health. Another limitation that needs to be acknowledged is the absence of patients’ perspectives in the current research. SPI is a relational practice, and patients’ perspectives on completing SPI, as well as the relationship and rapport built with their ED providers, can help explain the usefulness, completeness, or overall quality of SPI across both modalities. As such, we plan to work with patient partners in the next phase of this work to support the implementation of the Hope app.

Lastly, this study was an implementation needs assessment focused on clinicians’ attitudes, knowledge, and the environmental factors shaping clinicians’ delivery of the Hope app in the ED. As such, other implementation outcomes, such as fidelity, implementation cost, and maintenance, were beyond the scope of this project. Fidelity, in particular, assesses whether an intervention is delivered as intended [30], in this case, whether safety planning is completed in alignment with the Stanley and Brown model. While fidelity was not directly assessed in this study, our interview findings provided some insight into variation in SPI delivery, including differences in the time clinicians spent completing the SPI. However, we did not examine whether all five components of the safety plan were completed or how consistently they were completed. Assessing fidelity will be an important area for future research in the current study setting.

## Conclusion

Our study, grounded in behavioral change theory, identified key behavioral factors influencing the implementation of an app to support SPI in the psychiatric ED. These factors encompassed barriers and facilitators that collectively impacted clinicians’ capability, opportunity, and motivation. It was evident that tailored strategies were essential to address these barriers, while leveraging facilitators could help support and sustain future implementation efforts. In addition to the identified behavioral barriers and facilitators, our study highlighted the importance of ongoing monitoring and being open to adaptation of implementation strategies. By remaining attentive to evolving clinician needs and organizational dynamics, new barriers could arise, or facilitators might weaken, thus impacting the sustained use of the app in the ED.

## Supporting information

S1 File Safety plan. (DOCX)

S2 File Semi-structured interview guide. (DOCX)

S3 Table Expanded barriers and facilitators. (DOCX)
