# Supplementary material for: Barriers and facilitators to using an app-based tool for suicide safety planning in a psychiatric emergency department: A qualitative descriptive study using the Theoretical Domains Framework and COM-B model
Source: PLoS One. 2026 Oct 13;21(10):e0355517. doi: 10.1371/journal.pone.0355517 (PMC13660875; doi:10.1371/journal.pone.0355517)
Supplement: Supplementary file 1 [file pone.0355517.s001.docx]

Additional file 1. Safety plan

Paper version

The following safety plan template is adapted from Stanley and Brown’s 2012 paper entitled “Safety Planning Intervention: A Brief Intervention to Mitigate Risk for Suicide” and the CAMH Suicide Prevention and Assessment Handbook (2011).

**SAFETY PLAN**

**Step 1: Warning signs that I may not be safe**

1.

2.

3.

**Step 2: Remind myself of my reasons for living**

1.

2.

3.

**Step 3: Coping strategies that I use to distract myself or feel better**

1.

2.

3.

**Step 4: Social situations and people that can help distract me**

1.

2.

3.

**Step 5: People who I can ask for help**

1.

2.

3.

**Step 6: Professionals or agencies I can contact during a crisis**

1.

2.

3.

4.

5.

**Step 7: Making my environment safe**

1.

2.

3.

App version

| **Home page**  **Contents:**   - Safety Plan - Community Resources (curated) - Wellness Activities (e.g., Journaling, Grounding technique) - General Information (e.g., Causes and risk factors for suicide) - Crisis Line | **Core Component**: Safety Plan  Note: Safety plan includes 5 items | Example View 1  Item 1: My Warning Signs | Example View 2  Item 2: My Reasons for Living |
| --- | --- | --- | --- |
|  |  |  |  |
