# Supplementary material for: Barriers and facilitators to using an app-based tool for suicide safety planning in a psychiatric emergency department: A qualitative descriptive study using the Theoretical Domains Framework and COM-B model
Source: PLoS One. 2026 Oct 13;21(10):e0355517. doi: 10.1371/journal.pone.0355517 (PMC13660875; doi:10.1371/journal.pone.0355517)
Supplement: Supplementary file 2 [file pone.0355517.s002.docx]

# Additional File 2. Semi-structured Interview Guide

| COM-B | TDF component | Interview prompts |
| --- | --- | --- |
| Capability | Knowledge  Skills  Memory, attention and decision processes  Behavioural regulation | 1. Tell me about what you know about the Hope app and other ICT-based interventions for suicide prevention. 2. Tell me about your experience of delivering the Hope app (or paper-based safe plan) in the psychiatric emergency department. 3. What difficulties did you experience when providing the Hope app (or paper-based safe plan)? (Prompt: patients with low digital literacy, patients without smartphones) 4. What sorts of things (e.g., training) would help support or hinder you when delivering the Hope app to patients? 5. When do (would) you forget? / What would help make it easy to remember? 6. What do you think is needed to ensure your experience of providing app-based interventions at psychiatric emergency is easy? 7. What skills do you think providers need when delivering the Hope app as a means to deliver SPl? (Prompt: digital equity) |
| Motivation | Social/Professional Role and Identity  Beliefs about Capabilities  Optimism  Beliefs about Consequences  Reinforcement  Intentions  Goals  Emotions | 8. Tell me about your professional responsibility in providing care in relation to suicide prevention (or SPI) and providing the Hope app to patients? 9. How easy or difficult is integrate the Hope app into your routine clinical practice? 10. How confident do you feel in your ability deliver an SPI via mobile app to patients? (Any problems encountered?) 11. Why do you think the Hope app is/is not useful? 12. Are there any harms that have occurred due to the traditional paper-based SPI or any harms in using the Hope app? (Prompt: digital equity) 13. How optimistic/skeptical are you that we are moving away from traditional paper-based SPI and shifting toward integrating technology such as the Hope app? 14. Why do you think we need the new innovation for SPI such as the Hope app? 15. Do you have any anticipation for integrating the Hope app into routine practice? |
| Opportunity | Environmental context and resources  Social influences | 16. Do you have any existing resources that can be leveraged to facilitate the Hope app integration? 17. Are there competing tasks or time constraints that would influence your ability to deliver the Hope app to patients? 18. We talked about delivering the Hope app, how do you think this new innovation would fit in to the CAMH psychiatric emergency and the CAMH as a whole? 19. How do you think others will respond to delivering Hope app to patients? |
|  | App-specific* | 20. How easy for you to use the Hope app? What feature of the app helps/hinders the delivery to patients? |
| * A new addition based on the scoping review findings | | |
