# Supplementary material for: Barriers and facilitators to using an app-based tool for suicide safety planning in a psychiatric emergency department: A qualitative descriptive study using the Theoretical Domains Framework and COM-B model
Source: PLoS One. 2026 Oct 13;21(10):e0355517. doi: 10.1371/journal.pone.0355517 (PMC13660875; doi:10.1371/journal.pone.0355517)
Supplement: Supplementary file 3 [file pone.0355517.s003.docx]

Additional File 3. Expanded barriers and facilitators to using the app to complete SPI in the ED

| Domain | TDF | Theme | Subtheme | Barrier (B) / Facilitator (F)/ Mixed (M) | Example Quotes |
| --- | --- | --- | --- | --- | --- |
| Capability | Knowledge | Limited awareness of the Hope app* |  | B | "So what I know is, it's a free phone app that anyone can download. And there's some tools on it. Like suicide safety planning. And I believe there's breathing exercises, grounding exercises, and perhaps some maybe quick phone numbers for the hotlines. That's from my memory." [011]  "I don’t know much about them. I think this is the first I’ve actually ever heard of an app used for safety planning, like the type of safety planning that we use in the emerg. " [012]  "I didn’t know anything about it until I read your email. But I all I know is that it’s related to creating safety planning for patients. " [016] |
|  | Knowledge and skills | Demonstrated knowledge and skills for safety planning using the paper version |  | F | "Safety planning, the purpose of that used in the emergency is to understand the client and their individual needs and what we can do to prevent further safety risks. That allows the clients to be more involved in their own care as well." [004]  "What the warning signs are, coping strategies. What their support system looks like. How they can keep themselves safe, their reasons for living. What they could do, what they're in crisis, and who they can reach out to when they're in crisis" [013] |
|  | Memory attention and decision making | Decision making and prioritization of tasks including safety planning |  | M | "I'm supervising a whole bunch of residents who have urgent issues to tell me about. I also need to be available for the nurses. And then I have to also be seeing patients on top of that. I get limited in how much I can get done or what I need to prioritize." [026]  "If they were willing to get the app, it'd be easier to click through the options versus sitting with them and trying to explain a physical paper making them write it out. But I think it would really depend on the population. You have a teenager that would probably be fine. I'll download an app and then just click through it. That would be faster than sitting with them with a paper, but if you had maybe somebody that's not as familiar with technology and doesn't use their phone very often that doesn't know how to even connect to Wi-Fi, then trying to download an app and explain it, wouldn’t it be practical for them? But if there was the either or option of either the app or the paper and not both, and I think depending on the client, it could be easier or harder " [028]  "It's like a tool, right, it's like a toolbox. You open your toolbox and use a special tool for whatever you need, right. It's not a universal obligation for you. As a professional you figure OK, for this individual, this might work for this individual who's probably not be able to access this app or access email if somebody, some person lives in a shelter, who has no phone, has no laptop or tablet, maybe has no email address either, probably paper copy will be better to keep on them. But majority of people probably should be able to utilise this app." [002] |
|  |  | Remembering the app especially in the beginning phase of change |  | B | "I think, to be honest, we see so many random posters and signs everywhere, it's easy to just kind of tune them out." [003] |
|  | Behavioural regulation | Opting for the quickest way, and breaking habit |  | B | "I can see how, if staff is busy, if you have eight patients you need to run between, it's easier to hand the patient here, a paper form and say, “Here, do it” rather than a patient be coming back to you, “How do I do this? How do I download it? My Wi-Fi is not working.” Yeah, I can see how it can be a barrier, but with technology improving, maybe Wi-Fi gets more stable here. Because quite often, they ask you for a password and all that stuff and password expires." [002] |
| Motivation | Belief about capabilities | Level of technical confidence in using the Hope app |  | F | "I don't have any problem with the technology of it. I think if I were to put in the time and the effort, then absolutely, it could work." [009]  "I have helped people download it" [012] "For me very easy. I think it's not confusing at all. " [012]  "Right now, if I were to a scale of zero to 10 in confidence, I'd say maybe a seven. And that's only because I don't even know what the app is. I've never seen the interface" [004] |
|  | Belief about consequences* | Perceived level of workload changes and difficulty in using the Hope app |  | M | "I think it might be actually easier, sometimes, because we don’t have to run back, like, physically run back and forth to, like, grab the paper, get the pencil. And we can just, hey, like, try using this. Like, it’s pretty self-explanatory – you can spend time using it and you can just click" [016]  "No, I don't think that's a huge workflow increase, no. Workload increase, no." [007]  "I think it requires more time to actually move from talking about the safety plan to completing it. Because you've introduced the idea of it. And then the nation is – first of all, they need to get their phones, sometimes they don't have their phones on them, then they have to connect to the Wi-Fi, and that's kind of a pain sometimes. Then they look for the app, they download the app, that can take some time. And then it's kind of like, for that whole interaction, the clinician is just kind of sitting there waiting, right. And it doesn't really make sense for the clinician to leave and then come back in a few minutes. Because that's kind of an awkward amount of time, where it doesn't really give them an opportunity to work on anything else" [003]  "I think the practice change is going to be relatively easier. Because from my perspective, it just comes across, like I said, of having a tool on hand in my pocket, that will actually help me explain a safety plan better. And then I still have the option of offering the paper copy, which I generally offer anyways. The only thing is it requires more time." [003]  "I think we are technology savvy enough for each team member to if they need to sit down and show the patient how to use it. But that also takes time and that’s the thing that’s in short demand in the [ED]. So everybody is just so busy. So initially, just kind of sitting down and going through with patients how to use it, I can imagine staff members will feel like that’s another burden on their time. " [017]  "I think it might create new workload, in the sense of you might have people have, staff having to help patients figure out, how to download the app, how to open the app. And I think staff might see it as a more time consuming task." [009]  "I think it'll be hard, just because the idea of paper safety planning is so ingrained, and I think it's been going on for so many years... ... I think just when you get down to it, it's not that much effort, I suppose to download an app and open it up. But I think in the perception of people who are very busy, nursing is always so busy in the emerge, they might see all the extra step of you have to get the patient to download the app, then you have to open me up and presumably you'd have to check the app after they're finished, to read that they've done the safety plan. Just at a glance, it would seem a lot more work. " [009]  " I feel that it's a practical tool and the steps that it takes to put it on our phone and review it, it's not too heavy, it's not too intensive. It presents nicely, I don't think it would be difficult." [012]  "If, let's say you're the client, you come in, your phone was never used, or you don't know how to connect to Wi-Fi. Now I have to take away maybe five, 10 minutes, sit down with you, connect it with Wi-Fi. If you're unfamiliar with Wi-Fi, I have to explain that to you. And in that situation, I think you would probably give me your phone, I would do everything and just give it back to, and you have the app and you do it. That interaction itself would be five, 10 minutes" [004]  "I think when it comes down to time, not everyone has the time to actually sit down for – I guess, a [unintelligible] amount of time to be able to – that would be a situation where I feel like you’re teaching a patient as well as trying to... maybe just assist them in filling out this information. And I feel like that could double the time that it could take to actually just fill it out if it was just paper. " [025] |
|  |  | Uncertain benefits of safety planning beyond discharge |  | B | "Or maybe their situational factors are just too overwhelming for them in the moments where they presented, to the point where they weren't really able to use the safety plans. In those kinds of cases, trying to engage patients in a pen and paper plan felt more like a formality. And it felt like I was doing it partially because of a responsibility or a checkbox, and less because I really thought it would change the trajectory of care for the patient" [003]  "It’s honestly hard because if it’s successful, like, a lot of these people might not even come back to the ED. " [021]  "It would be great to survey people who have actually downloaded the app, whether or not they find it useful. I don't know. Because I never been in that sort of emotional predicament where I'm needing a safety plan for myself. So I don't know whether it's useful. I mean, objectively, it looks like it should be useful. But I don't know whether we have feedback from end users" [007] |
|  |  | Perceived benefit and disadvantages of the Hope app |  | M | "I think the other thing is, as I mentioned, having those toggles of just, oh, yes, this applies to me, this applies to me, might make it a little bit more passive." [009]  "People are really excited about it. Patients are really onboard." [024]  "The paper you can leave in your car, in your home, in your bag and or lose it and eventually – with the app, you can always open it up and then remind yourself it's always with you, if you want it, instead of trying, OK, I left my safety plan at home. But now at my parents’ house, I don’t feel safe, I feel distressed and tried to remember what I did. And this way I have it on you. I think that's a big advantage." [002]  "I do support going paperless, it's just tough, I think. Then also patients can easily lose that, there is a benefit to the app in the sense that those who have phones have their phones with them all the time. The app – I think there's obviously some people who don't have phones or can't figure out their smartphones." [009]  " I feel the app is really great, because it's just easier to have in one place, especially when they're on their phone, and they can send it directly to whoever right away. And sometimes it's just helps. It's more than that. It's better than writing because sometimes they can get so stressed out that they get fed up and they don't want to write something" [010]  "I think it's so great that they can carry it with them when they leave. Because people –especially people, they don't care about paper like that. They'll throw it out, but on their phone, they can take it everywhere. " [010]  "When we're cleaning the rooms, we see so many safety plans left behind." [010]  " It's a tool that a client can access wherever they are people, more than likely always have their phone with them. A client can access it no matter where they are and at any time, versus the paper document that probably gets shoved in a drawer. And the client will forget about and not take seriously. At least it's something that can be accessed anywhere. And that's helpful for the team to remind the person, the client that this safety plan is with them at all times. And it's instantly accessible. I think it just helps the team because it helps us reassure the client that the plan is with them at all times." [012]  "I mean, when would they use it again? Only in moments of crisis, right. And in moments of crisis, they would be here. I think if this was more scheduled somehow as a weekly check in. If you guys could navigate for every so often, depending how high the safety risk, just have the app check in on them and see, hey, are these still your safety plans? Do you still feel the same way? Because if this is a one moment of the thing, it doesn't really serve too much purpose for the individual, more so for the clinician." [003]  "I think it's useful, more mostly in the clinician’s perspective, as opposed to the patient's perspective. And that's only because, like I mentioned that piece of paper, once we've given it to them, more times than not, they just throw it out, it doesn't actually mean too much to them. I think out of 10 patients, maybe three or four would actually use that as the set purpose, the safety plan." [003]  " I will say the biggest thing is patients really like it. And like I said, a lot more than they like the paper. Not that they ever minded the paper, but they really like [the app], and they really like having it on their phone and being able to open it easily and it feels like, I think, like this comforting thing that you can't lose the way that you would immediately lose the paper. And that it’s a working document and that you can change it" [024]  "So the app will increase client engagement, because it allows them, it's kind of like the paper, the paper is also allowing the client to independently be the focus of the care. It's different when I tell you, “Hey, these are the resources. Use them if you want.” Versus you telling me this is my personal one, because what works for you, won’t work for the next person. So the app is just an extension of that, which allows them to do their individual unique safety plan, which is different than other persons."[004] |
|  |  | Anticipation of patients' reactions for checking the phone (Reading over patient's phone may not be well-received) |  | B | "Looking over somebody's phone is invasive a little bit? Yeah, a little bit. And not everyone might be comfortable to do that or to hold the phone itself or with COVID, germs." [012]  "I think it makes it a little bit difficult in the sense that I'd have to go back in and see the patient and read their phone. Whereas in this scenario, it's usually nursing brings me back the worksheet that the patient has completed. And then I just review it, copy it, give the original back to nursing so they can give back to the patient or give back to the patient myself." [009]  "Yeah, for me, the only thing I don't like is it sometimes I'd like to write…somebody will write the safety plan with a person. So they'll tell me and I'll write it up for them. So I don't know if I would grab a person's phone and, start typing on their phone, necessarily." [027] |
|  |  | Beliefs about equity with regard to the Hope app |  | M | "My grandfather has a cell phone, but he'd rather write something down and type it into a phone. He doesn't even probably know how to type a text message. So it's nice to have it accessible. So, I mean, the phone helps for younger folks or people that are interested in technology. But that's not the majority, right, unfortunately. Well yeah, we see a lot of homeless people, impoverished people come in with police, they don't have cell phones." [005]  "There might be some people who are not very good with English, they might need extra help. And also I’m just assuming this, but the app is probably just in English, right?" [006]  "If that patient then decides to come in and use our tablet, because they don't have a phone. And now it's out in the either in the internet on a server, but they don't have access to technology. If they wanted to send that, where do they go to access technology, when they send it, to access their email? I've met a lot of people that have emails, but no means to access it.” [005]  "I think using cellphone for the population out there is feasible just because smartphone are so – they are everywhere." [020] |
|  | Goal | Safety planning is high priority for ED care |  | F | "The purpose of [safety plan] used in the emergency is to understand the client and their individual needs and what we can do to prevent further safety risks. That allows the clients to be more involved in their own care as well." [004]  "I just think that at the end of the day, it's promoting safety, right. So we promote mental health in a way that is not like this daunting cloud? It’s something that you can work towards? Yeah, you're having a rough day, but at the same time, there are ways that you can work towards having a better day tomorrow and the safety plan, is that" [010]  "Safety planning in the emergency department. It is always at kind of like top" [018]  "Usually it's a requirement for somebody that has depression or suicide. For some sort of history of self harming. Then for sure that they require, a complete safety plan, they can’t even be discharged. So, can't really think of instances that I've seen where it's been absolutely missed" [028]  "If there's anything particular – if it's chaotic, sure, it might get missed. But when it's determined by the doctors that a safety plan is required, we have to complete it. ...I don't see the doctors not using it when they clinically determine, OK, we're going to need to do a safety plan for the patient, then that has to be done" [012] |
|  | Professional responsibility* | Professional responsibility for safety planning |  | F | "I think it comes from discussions with your team, and the feedback that you get from each other. And reminding each other of opportunities to have a safety plan be discussed. I know that's a little bit vague, but I think it comes with a cultural shift, where we eventually start thinking of safety planning as a therapeutic tool and incorporate that as a focus of our plan for a patient, as opposed to just something that we do before discharge." [003]  "I think it’s very important. Like it’s one of our – it should be one of our key assessments that we do as nurses here. Because even though the doctor, you know, is mainly the one who makes the decision about a disposition for a patient, our value as nurses is most of the time like taken into account by the doctors." [012]  " I don't think it should be just a nursing task. I think the psychiatrists could mention [the Hope app]. The social workers could mention it. Nurses could mention it. And even the program assisted staff could mention it. I don't think this is strictly nursing tasks." [014]  "I think safety is all of our responsibility. And the Hope app supports patient safety." [015]  "I think it's very feasible. Just about making sure that we do it. And how to you make sure well, we have team meetings, social work team meeting. So if we say that this is a goal, we have, seasonal meetings. So at that next meeting, if we say, let's make sure we do this for each client, it's going to be on our to do list." [012] |
|  |  |  | Medical legal | M | "I don't know if I if they did the hope app. I probably would just say they complete the hope app. And I eyeballed it looks good. And then if there was an adverse outcome, that's all the information we would have, but I think that would be enough for the safety committee. And they would know they could pull up what is the Hope app? The recent version was that look like what would the client have filled? I think that would have been enough medical legally." [027] |
|  |  |  | Shared responsibility to support the Hope app implementation including tech support | F | "[Tech support is] something that we can all do. Because if we are familiar with the app, and we know how to use it, we should be OK" [010]  "we also have other people that can help with some of those things like PAs can help with, with setting up the Internet, they can help them to open the app. And then if they need any more help, they can ask the nurses, so it's not solely on the nursing staff." [013] |
|  |  |  | Variability in how clinicians approach safety planning related to time spent with patients | M | "There are definitely certain days where there's no time, but I think you have to make time. Especially for these vulnerable patients, right. And for me, personally, my practice, I don't – especially these patients that are going through depressive episodes or have suicidal ideation, I never want them to think that I kind of abandon them. So for – the safety planning takes 10 minutes of your time, right, maybe a little bit more. But I don't know, I think it's, I think this is a nurse to nurse question. And everyone's practice is different. But I personally, even if I'm really, really busy, I will try to make time for these people." [014] |
|  |  | SPI is important professional role for all disciplines in the ED |  | F | "My role is to help people safety plan, but also to give them some sort of independence to safety plan. I'm not going to give them the answers, I will work with them to get the answers. But yeah ultimately, I really try to encourage people to safety plan by themselves, because nurses aren't going to be there with you forever, right. Patients also have to be self-sufficient, right. And one of the things that's really important about safety planning is that you don't coddle patients, you let them have their independence and do it themselves. I think that's really important." [14]  "I think emergency nurses are special because they work with patients for a brief period of time but they have to build the relationship really quickly and probably spend more time with patients than the physicians do. So nurses are really well positioned to take the lead on supporting patients with the Hope app. " [015]  "I’m working here, like an ED, I feel like anyone that comes to seek for help, I feel obligated to do my best to keep them safe. And just because they come at the lowest moment of their life and, I’m able to help – that’s great. But if I don’t do enough then, I question myself, you could have done better. Like, you could have spent more time with that person. Or just because I was preoccupied with something else and I couldn’t spend more time with that person, that person could have gotten better care. So I feel like safety planning is part of me doing my obligation in by sitting with them and just go through the questions with them. " [016]  "Not to stop using paper for people who prefer it or don't have another option. And to still provide that. Like again, the process of the safety plan and the mental process of working through it is a lot of the intervention, so even if they do lose it, I do believe that a lot of people that way at least can generate it again. So, it still was helpful. "[024]  "Safety planning in general. I mean, I think that that's part of our assessments, that's really the sort of bread and butter of what we do in the emergency department" [007]  "Yeah, I just – I mean, safety planning is really important, right? We don't like to leave – we don't like to let people leave hospital if they're not really to safety plan. And as a nurse, especially as a mental health nurse, I feel it's my due diligence to safety plan, and the Hope app is something that they'll always have with them. So yeah, it's a really good way to Safety Plan." [014] |
|  | Optimism/  pessimism | Personal preference towards digitizing healthcare services including safety planning |  | M | "If it’s not integrated with the health record, we would prefer paper because paper gets stand in." [015]  "I even myself, prefer writing, like, on the paper." [016  "For me, the only thing I don't like is it sometimes I'd like to write, like, I'll write like, somebody will write the safety plan with a person" [027]  "I'm getting kind of tired with technology. And so maybe like what resistance do I have as a, I think my biggest resistance is probably paper to me feels a bit more therapeutic than a computer screen. Also, given the documentation doesn't get into the charts. So it doesn't feel as it might be more worthwhile for the patient. But from a clinician perspective, having a in the chart to me is very important." [027] |
| Opportunity | Social opportunity | Communication for safety planning |  | B | "There's suicide risk assessment and suicide evaluation. But there's no checkbox for was a safety plan administered? Or should we do it? It's just case by case? We feel in the moment that clinically it's a necessary tool, then we'll, the doctor will say let's do a safety plan or the social worker would say let's do a safety plan. Then we do it and then review it." [012]  "In my experience, every time a safety plan needs to be done the doctor has told me, “Can you just make sure they fill out a safety plan before they leave?” Other than that time I think it would be based on the nurse’s judgement whether or whether or not the safety plan should be filled out. That’s actually a grey area for me. I'm actually not too sure if it’s every time the doctor says to do one, or if it's based on also if we think that the patient needs to do one. That's a grey area for me." [025] |
|  |  | Team culture in the ED |  | F | I found the team is really cohesive in the emerge. It’s a really great team to work with. [024]  "Communication is important. And our team does that well. And we support each other outside of our roles, because we're family, so. And we try to pass that on to the patients for sure. And once we acclimate ourselves, I don't see any problems rolling it out." [005]  "With this new practice change, we have to do, take some time, people get used to it, but then once you're used to it, it's fine." [028] |
|  | Physical opportunity | Lack of access to smart devices among subgroup of ED patient population |  | B | "What I'm saying if that patient then decides to come in and use our tablet, because they don't have a phone. And now it's out in the ether in the internet on a server, but they don't have access to technology. If they wanted to send that, where do they go to access technology, when they send it, to access their email? I've met a lot of people that have emails, but no means to access it." [005]  "I think a lot of our clients who are homeless, living in shelter, they're chronically getting their items stolen because of the culture in shelters so many of our clients might get one phone and it's gone in a week. Or with people with chronic addiction, they're selling their phones. " [012]  "I think the barrier, from my perspective, is that is that is that people need to have a smart, smart device. Yeah. Or phone. I think that's, I know that probably most people do now, but we also do work with people who are experiencing homelessness or poverty. So not everybody has access to that. And then and then like, like, you know, and like I said earlier, like people can have technological challenges, which again, we can help problem solve. But sometimes people just don't like to use apps and that's a preference thing" [029] |
|  |  | Busy ED setting and being understaffed, need for prioritizing multiple tasks |  | B | "But I think during very peak periods of busyness, they won't use it, because they forget, or it will just be too though they'll opt for like the quickest thing to do, which would be like grabbing a piece of paper." [001]  "And I feel like it’s something that shouldn’t be rushed but it ends up being rushed because we – like, even today we have, like, five, six discharges happening at the same time. And if that’s the only thing that’s holding them back then, like, hey, as long as we have something on the paper then, like, you can go." [016] |
|  |  | Lack of documentation infrastructure in the EHR for the Hope app |  | B | "I think downloading an app should be fairly simple. I think most people in emerge would be fine with that. One other issue that I'm just now thinking about is that, as I mentioned, we like having the safety plan to put in the file. We like having – before I discharged someone, I like seeing that they filled out the safety plan. And if it's on their phone, I kind of wonder how I would be able to see that? Would I have to go and look on their phone in order to see that they filled things out? Because that sounds like it would be – it's a lot easier then for the patient to say they'll do it, and then not fill it out." [009]  "I think tools like a pop up reminder or a tab can be added to our multidisciplinary assessment." [011]  "But if it does become placed into the multidisciplinary assessment, which is the tool that we do for every emerge visit, if it's part of that standard checklist of things to do, then it's part of what we have to do so then it's not, it won't be overlooked." [012] |
|  |  | Wi-Fi and other technical resources in the ED |  | B | "I think the most challenging part is getting access to the Wi-Fi, because it’s like a long code to type. And then some patients, they can't see properly so then we’ll have to take their phones and then we’ll type it in for them. " [023]  “Yes, like I said, so if the patient was really eager to get discharged, telling they can stay safe. But I want to see a safety plan before they go. But now they're telling me, “Well, I can't seem to download it. The internet’s really spotty.” Then we're kind of at, like, a difficult situation where I technically, I don't have grounds to keep them. I can't really imprison them in the hospital to do this app. To finish this.”[026] |
|  |  | Organization culture - Alignment of the Hope app's purpose with the organizational value and goal |  | F | "I think it fits with the organisation in the sense that [the organization] is continually striving to innovate the way that care is provided. So as I mentioned, it makes sense as generations change and things become more digital." [009] |
|  |  | App-specific | Layout, usability, resources, | F | "It's pretty straightforward, I think that's what matters. And like I said before, it's, multiple choice is always easier than to come up with something, less time consuming" [002]  "Yeah, it's pretty user friendly," [007] |
|  |  |  | Lack of translation function in the app | B | "The multi language could be useful. Because that eliminates the needs of getting it translated and all that stuff." [020]  "I noticed there are a lot of language barriers as well. So, maybe just having an option to translate the page or the app. Or I don't know if that's a feature that will be with the phone. Like, if the phone is set to French will the app be installed with French language built into it?" [023] |
|  |  |  | Toggle options in the safety plan | M | "I think one of the challenges that I've encountered is, depending on how engaged the patient is with the process of coming up with a safety plan, and what their mental status is, they may have difficulty coming up with items that they can put on the list. For example, someone presenting in crisis might say something like, “Oh, I have no one supportive in my life.” And they would have a really hard time coming up with resources or people or clinical team members who would be able to support them if they were ever in crisis in the future. So in a way, I think having options that are presented to them, can be really valuable." [003]  "The one other thing that I was just thinking of, I like that you have the little toggles to decide, if that applies to them, and then at the end, it's optional to write in. Actually kind of feel like it's sometimes beneficial that on the worksheet, it's totally blank. And they have to think and write it in. Whereas they feel like there might be a passive kind of just, yeah, sure, sure, like checking off a bunch of ones. And it's hard to tell then if the safety planning was really thought through." [009]  "I think the other thing that actually is a con of the app, in my opinion, is actually having the choices there may make the clients, it might be easier for them to not really actually think about it, if that makes sense. Like we're actually doing a paper form, even though because there's no options, they have to come up with them. So it's more spontaneous, they actually have to think about it. Whereas this app, there is a small risk. I think it's small, but a risk that like you, or I could just click on the thing, and then just toggle whatever we think and even if we're not really processing, like, do we actually mean that? Does that actually apply for us? You know, yeah, so that's the only other risk I see."[029] |
|  | Social opportunity | Leadership support for change management |  | F | "I think that people, if you make it mandatory, and you make all of us attend, I think that'll be easier than a sticker on – you can still have the poster on the wall."[010]  "It’s called ED Communications. And then in that email, there’s always like a section about, this is a change in practice and then she kind of types it out, describes it. For example, if it was for the safety plan it would be like, you know, “Usually we do safety plan by paper but now we’re doing something new using the app.” "[012]  "I don't really have anything to add to that, because our team is pretty good. And if [leadership team] asks us to implement something in our practice, I think everyone is pretty good about it." [014]  "I think if we can, you know, get people on board with it through leadership, like, endorsing it, through physician leadership endorsing it, some champions, maybe, or whatever we want to call them, then yeah, I think it could work well. But it will take time. Practice change takes a lot of time." [015] |
|  |  | Patients' readiness for safety planning |  | B | “Sometimes patients who don't want to be discharged, they will sabotage the discharge by refusing to participate in the safety plan or so on. I think that's the biggest challenge sometimes, if someone doesn't want to leave, and the doctor insists on leaving, and a part of the discharge, conditional being discharged, completing a safety plan. So that could be a challenge.” [002]  " There’s been instances where they, for example, just say that there’s nothing. Like they can't fill it out at all, they have nothing to write. And that becomes a little bit tricky to kind of persuade them. Especially when we're really rushed in the emerge and there’s so many other clients to be seen. It can be difficult to kind of engage them meaningfully." [024] |
|  |  | Patient characteristics in the ED  (including age, preference, mental health conditions) |  | M | "The phone helps for younger folks or people that are interested in technology. But that's not the majority, right, unfortunately." [005]  "I got to say, even elderly people with cognitive impairment, if they have a smart phone, this is not a problematic app to use. It is a short download, people find it, they find it easy to use. If they have a smart phone." [024]  "I think it depends on the age range. I found with younger clients that are in their teenage years or maybe a bit more reluctant to fill out a physical paper or early twenties they don't want to do it. I'd rather scroll on their phone. So I think the app might actually be a solution to that. It'd be easier to kind of click through the questions and type it versus writing on a physical paper that they're going to lose right away." [028] |
|  |  | Alignment of the app's purpose and ED and organizational goal/vision |  | F | "I think [the organization] is, is really great like that we were always sort of ahead of the curve, I think. And when people come here, they're always very impressed by all the technology and all the new things that we do. Everything like that. So that's pretty cool."[013] |
| * Means salient themes | | | | | |
